# The count of tender rather than swollen joints correlates with aortic stiffness in patients with rheumatoid arthritis

**DOI:** 10.1186/s40064-016-2066-z

**Published:** 2016-04-11

**Authors:** Konstantinos Triantafyllias, Michele De Blasi, Isabell Hoffmann, Thomas Thomaidis, Philipp Drees, Andreas Schwarting

**Affiliations:** ACURA Rheumatology Clinics, Kaiser-Wilhelm-Str. 9-11, 55543 Bad Kreuznach, Germany; Institute of Medical Biostatistics, Epidemiology and Informatics (IMBEI), Johannes Gutenberg University, Mainz, Germany; First Department of Medicine, Johannes Gutenberg University, Mainz, Germany; Department of Orthopaedics and Trauma Surgery, Johannes Gutenberg University, Mainz, Germany

**Keywords:** Rheumatoid arthritis, Aortic stiffness, Joint tenderness, Pain, Cardiovascular risk, Carotid-femoral pulse wave velocity

## Abstract

**Background:**

Patients with rheumatoid arthritis (RA) are at a higher cardiovascular (CV) risk in comparison to the general population. CV risk associates closely with aortic stiffness. Aim of this exploration was therefore to evaluate aortic stiffness in patients with RA and to examine its association with various RA associated parameters as well as with traditional CV risk factors.

**Methods:**

Measurements of carotid-femoral pulse wave velocity (cfPWV) were analyzed retrospectively in 38 RA patients and 25 controls. We investigated the statistical difference between cfPWV values in the two groups. Furthermore, we analyzed the associations of cfPWV with laboratory and clinical RA parameters including Disease Activity Score 28 and its components, rheumatoid factor, cyclic citrullinated peptide antibodies, antinuclear antibodies and RA duration. Finally, we explored the relationship of cfPWV with traditional CV risk factors in the RA group.

**Results:**

cfPWV was not significantly higher in RA patients in comparison to controls in an adjusted statistical model for confounding factors [−0.587 95 % CI (−1.38 to 0.201), *p* = 0.144]. Among RA patients there was a statistically significant correlation of cfPWV with age (rho = 0.544, *p* = 0.001) and the count of tender joints [0.051 95 % CI (0.008–0.207), *p* = 0.034]. Finally, C-reactive protein associated only marginally with cfPWV [0.105 95 % CI (−0.410 to 0.003), *p* = 0.053].

**Conclusions:**

In RA patients the number of tender, rather than swollen joints correlates with stiffness of the aorta, as measured through cfPWV. Therefore, RA associated joint pain might play a role in the development of aortic stiffness and thus increase CV risk.

## Background

Autoimmune diseases, such as rheumatoid arthritis (RA), correlate with high cardiovascular (CV) risk and high morbidity and mortality rates (Abou-Raya and Abou-Raya 2006). In particular, CV diseases are considered to be responsible for the cause of 40–50 % of all deaths in patients with rheumatoid arthritis (del Rincón et al. 2001). CV risk in the general population can be assessed through various markers, one of which is arterial stiffness. Particularly, stiffness of the aortic vasculature is a modifiable, independent predictor of CV risk and can be measured through carotid femoral pulse wave velocity (cfPWV) (Laurent et al. 2006). The predictive value of this marker concerning CV events has been shown in a series of epidemiological studies and cfPWV is nowadays characterized as the gold standard for the assessment of aortic stiffness (Laurent et al. 2006).

cfPWV gives information about the elasticity of the vascular bed among the carotid and the femoral artery (Baulmann et al. 2010). In contrast to parameters such as blood pressure, lipids or glucose which match the instantaneous intensity of traditional CV risk factors, cfPWV reflects the long-term effects of established and unknown risk factors together with the individual genetic predisposition of the patient (Vlachopoulos et al. 2014).

A number of studies reported a statistically significant increase of cfPWV in patients with RA in comparison to their healthy counter partners (Mäki-Petäjä et al. 2006; Kocabay et al. 2012; Turkyilmaz et al. 2013). However, some other studies came to the opposite conclusion (Stamatelopoulos et al. 2009; Arida et al. 2015). Moreover, the association between aortic stiffness and different RA associated activity and chronicity markers is still somewhat unclear. Aim of this study was therefore to test the hypothesis of increased aortic stiffness in a group of RA patients in comparison to healthy subjects and to examine whether there is a correlation of cfPWV with various clinical and laboratory RA associated parameters.

## Methods

We analyzed retrospectively routine measurements of carotid-femoral pulse wave velocity in 38 patients with RA and 25 subjects without systemic rheumatic or CV diseases. The measurements were conducted in our inpatient rheumatology clinic in Bad Kreuznach, Germany as a part of the diagnostic process. Patients with malignancy, pregnancy, age <18 years, active infection and CV disease were excluded from the analysis.

All patients with RA met the 2010 American Rheumatism Association (ACR)/European League against Rheumatism (EULAR) classification criteria for RA.

### Arterial stiffness measurements

The examination protocol of cfPWV was in accordance with the recommendations of the expert consensus document on arterial stiffness (Laurent et al. 2006). cfPWV was measured as the velocity value calculated through the distance between the carotid and femoral artery in meters (m) divided by the time that one pulse wave needs to cover this distance in seconds (Δs/Δt) (m/s). All of the measures were conducted from the author and two experienced independent medical assistants using a validated non-invasive device (Vicorder^®^, SMT medical GmbH&Co).

cfPWV was assessed three consecutive times in every patient and the average value of the measures was documented and used in the statistical analysis.

During the procedure, a neck pad was placed around the neck of the patient. The pad contained a small bladder which was placed over the carotid artery. A cuff (similar to blood pressure cuffs) was then strapped at the thigh of the patient. The bladder of the neck pad and the cuff inflated as the test started. After deflation pressure waves from the carotid and the femoral artery appeared on the screen of a connected laptop. The waves were then recorded simultaneously and the time delay between carotid and femoral wave was determined.

### Data collection

In addition to epidemiological data of both groups (gender, age), clinical and laboratory parameters of chronicity and activity in the RA group such as disease duration, disease activity score 28 (DAS28) as well as its components [C-reactive protein (CRP), count of tender (TJC) and swollen joints (SJC), visual analogue scale (VAS)] were examined. Values of ANA, rheumatoid factor and anti-CCP-antibodies were also recorded. Finally, the presence of traditional CV risk factors such as smoking, known arterial hypertension, obesity [Body Mass Index (BMI) > 25 kg/m^2^], hyperlipidemia and type II diabetes of the subjects of both groups were documented.

### Statistical analysis

The assumption of normality of distribution was evaluated through the Shapiro–Wilk test. Continuous variables were found to be skewed and therefore presented as median (25th and 75th percentiles). Categorical variables were summarized as absolute (n) and relative (%) frequencies.

The difference of cfPWV values between RA patients and controls was evaluated through Mann–Witney U test as cfPWV was not normally distributed. This test was also used to evaluate the association between cfPWV and categorical variables with two categories. In order to assess the correlation between cfPWV and continues characteristics, the Spearman correlation coefficient was used.

Furthermore, difference of cfPWV values between RA patients and controls after controlling for possible confounding factors was examined through an appropriate multivariate model. The same statistical model was used to check for the effect of confounding factors on the established correlations among patients with RA.

Descriptive statistics, regression analyses and tests have only been analysed by referring to cases without missing values. A probability value of 0.05 was considered statistically significant. We made no formal adjustment for the number of performed tests. Thus, the p-values should be considered to be exploratory. All statistical calculations were performed using the SPSS version 22.0 software (SPSS Inc, Chicago, Il, USA).

## Results

There was a similar distribution between patients with RA and controls regarding gender, arterial hypertension, obesity, diabetes mellitus and hyperlipidemia (Table 1). Patients with RA had a higher average age in comparison to the control individuals (*p* = 0.027) and the RA-group included more smokers than the control group (34.2 vs. 12 %, *p* = 0.041) (Table 1).

**Table 1:** Descriptive characteristics by group

	Control (n = 25)	RA (n = 38)	Significance (*p*)
Age (years)^a^	56 (42, 59.5)	61 (50.50–70.25)	0.027*
Gender (female)	21 (84.0 %)	32 (84.2 %)	0.982
Nicotine use (smokers)	3 (12.0 %)	13 (34.2 %)	0.041*
Arterial hypertension	12 (48.0 %)	18 (47.4 %)	0.960
Obesity (BMI > 25 kg/m^2^)	11 (44.0 %)	19 (50 %)	0.500
Hyperlipidemia	5 (20.0 %)	12 (31.6 %)	0.250
Diabetes mellitus (type II)	1 (4.0 %)	5 (13.2 %)	0.214
ANA positivity (>1:80)	–	5 (13.9 %)	–
BSG (mm/h)^a^	–	25 (14.00–55.50)	–
VAS (mm)^a^	–	7 (5.75–8.00)	–
CRP (mg/dL)^a^	–	1.1 (0.36–2.62)	–
DAS 28 (BSG)^a^	–	5.10 (4.10–5.75)	–
DAS 28 (CRP)^a^	–	4.50 (3.45–4.92)	–
Disease duration (years)^a^	–	11 (4.00–17.00)	–
RF (IU/ml)^a^	–	103 (6.55–322.50)	–
CCP (IU/ml)^a^	–	117 (7.25–340.00)	–
TJC^a^	–	4.50 (1.00–7.75)	–
SJC^a^	–	2 (0.00–4.00)	–

The rest of data are presented as absolute (n) and relative frequency (%)

*RA* rheumatoid arthritis, *ANA* antinuclear antibodies, *RF* rheumatoid factor, *Anti-CCP* cyclic citrullinated peptide antibodies, *DAS28* disease activity score 28, *CRP* C-reactive protein, *TJC* tender joints count, *SJC* swollen joints count

* *p* < 0.05

^a^Data are presented as median (inter quartile range) as they are not normally distributed

### Association between group (RA vs. control) and cfPWV

**Fig. 1:**
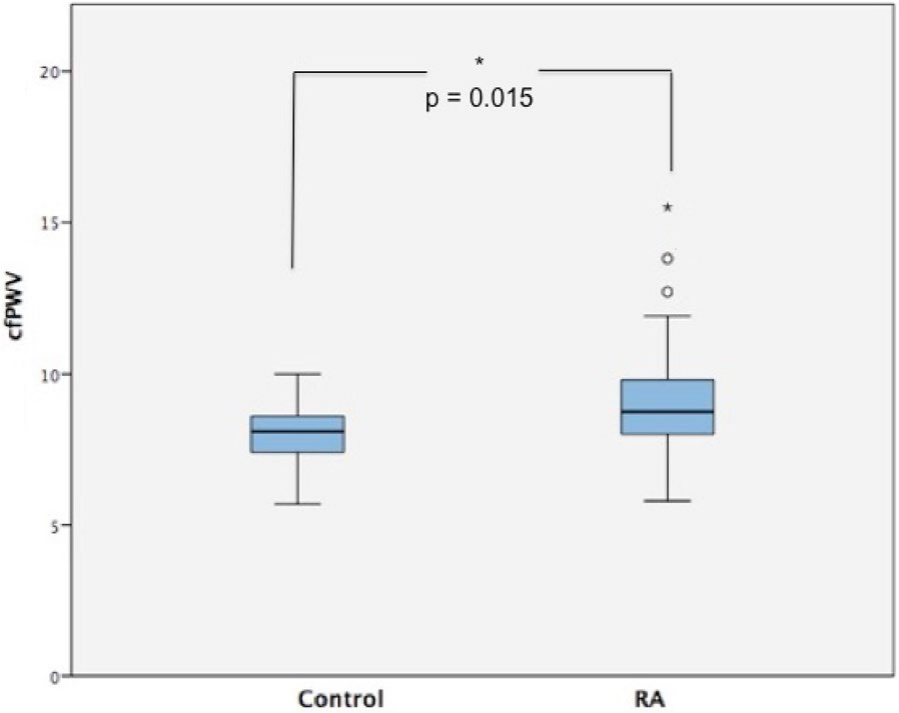
cfPWV in control and group RA. *RA* rheumatoid arthritis, *cfPWV* carotid-femoral pulse wave velocity. * *p* < 0.05

cfPWV average was found to be statistically significantly higher in the RA group compared to control group [8.75 (7.90–9.85) vs 8.10 (7.25–8.65) m/s, *p* = 0.015] (Fig. 1).

To adjust for factors which had a statistically different distribution in the control and RA group (age and nicotine use), and therefore could have a confounding effect on the results, multiple regressions were performed.

The age-adjusted statistical model revealed that cfPWV was only marginally higher in the RA group in comparison to the control group [−0.72 95 % CI (−1.50 to 0.04), *p* = 0.062]. Moreover, adjustment for both age and nicotine use revealed a non-significant statistical difference between cfPWV in the two groups [−0.587 95 % CI (−1.38 to 0.201), *p* = 0.144].

### Associations of cfPWV within RA-group

**Fig. 2:**
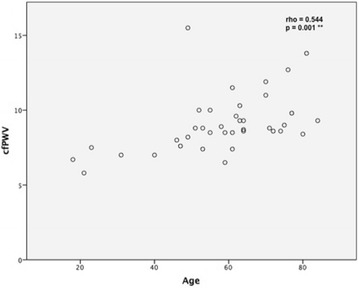
Association between cfPWV (m/s) and age (years) in RA patients. *RA* rheumatoid arthritis, *cfPWV* carotid-femoral pulse wave velocity. ** *p* < 0.01

Among patients with RA, it was found that cfPWV is significantly correlated with patients’ age and RF. This means that older patients have higher values of cfPWV (rho = 0.544, *p* = 0.001) (Table 2; Fig. 2) and higher RF levels are associated with higher cfPWV values (rho = 0.369, *p* = 0.025) (Table 2). Moreover, cfPWV was found to be marginally positively correlated with TJC (rho = 0.330, *p* = 0.065) and obesity [8.80 (8.50–10.30) vs. 8.60 (7.2–9.15) m/s, *p* = 0.071] (Tables 2, 3).

**Table 2:** Association between quantitative patients’ characteristics and cfPWV in RA patients

	Rho	Significance (*p*)
Age	0.544	0.001* (−)
Disease duration	0.021	0.903 (0.913)^‡^
CRP	−0.027	0.876 (0.053)^‡^
ESR	−0.031	0.859 (0.170)^‡^
RF	0.369	0.025*(0.462)^‡^
Anti-CCP	0.142	0.401 (0.345)^‡^
TJC	0.330	0.065 (0.034*)^‡^
SJC	−0.065	0.713 (0.744)^‡^
VAS	0.117	0.509 (0.763)^‡^
DAS28 (ESR)	0.191	0.331 (0.658)^‡^
DAS28 (CRP)	0.213	0.266 (0.499)^‡^

Spearman rho was used

*RA* rheumatoid arthritis, *CRP* C-reactive protein, *ESR* erythrocyte sedimentation rate, *RF* rheumatoid factor; *Anti-CCP* cyclic citrullinated peptide antibodies, *TJC* tender joints count, *SJC* swollen joints count, *VAS* visual analogue scale, *DAS28* disease activity score 28

* *p* < 0.05

^‡^in parentheses *p* values adjusted for age and obesity by multiple regression

**Table 3:** Associations between qualitative patients’ characteristics and cfPWV (m/s) in RA patients

	Median (25–75 percentiles)	Significance (*p*)
Gender		
Male	8.55 (8.20–8.93)	0.471 (0.598)^‡^
Female	8.80 (7.63–10.00)	
ANA		
Positive (>1:80)	8.70 (8.00–9.60)	0.993 (0.768)^‡^
Negative	8.80 (7.10–10.50)	
Nicotine		
Non-smokers	8.70 (7.66–9.75)	0.937 (0.617)^‡^
Smokers	8.70 (8.05–9.45)	
Arterial hypertension		
No	8.60 (7.00–9.30)	0.176 (0.059)^‡^
Yes	8.75 (8.50–10.00)	
Obesity (BMI > 25 kg/m^2^)		
Non-obese	8.60 (7.20–9.15)	0.071 (−)^‡^
Obese	8.80 (8.50–10.30)	
Hyperlipidemia		
No	8.75 (8.05–9.30)	0.867 (0.424)^‡^
Yes	8.90 (7.45–10.23)	
Diabetes mellitus (type II)		
No	8.60 (7.63–9.53)	0.182 (0.234)^‡^
Yes	9.30 (8.20–12.90)	

Mann–Witney U test was used

*RA* rheumatoid arthritis, *cfPWV* carotid-femoral pulse wave velocity, *ANA* antinuclear antibodies, *BMI* body mass index

^‡^ in parentheses p values adjusted for age and obesity by multiple regression

However, after adjusting for possible confounders (age, obesity) the association between RF and cfPWV did not remain statistically significant (*p* = 0.462) (Table 2). On the contrary, the statistical significance of the association between cfPWV and TJC increased [0.051 95 % CI (0.008–0.207), *p* = 0.034] (Table 2).

Control of all results through adjustment for the same confounding factors showed marginal correlations of cfPWV with CRP [0.105 95 % CI (−0.410 to 0.003), *p* = 0.053] and arterial hypertension [0.566 95 % CI (−0.039 to 2.179), *p* = 0.059]. Finally, gender, ANA positivity, nicotine use, hyperlipidemia, diabetes, disease duration, ESR, anti-CCP, SJC, VAS, DAS28 (ESR) and DAS28 (CRP) did not significantly correlate with cfPWV in the adjusted statistical models (all *p* > 0.05, Tables 2, 3).

## Conclusions

### Pain and aortic stiffness

The novel information of this exploration is the determined association between cfPWV and TJC in patients with RA. To our knowledge, there are no published studies in which the relationship between aortic stiffness and isolated parameters of the DAS28 in RA patients were investigated. Interestingly enough, cfPWV did not correlate with other parameters of disease activity such as SJC, VAS, ESR. The association between CRP and cfPWV was statistically marginal.

These results might reveal a closer association of cfPWV with joint tenderness and pain than with the acute joint-located inflammation in the course of arthritis.

It has been postulated that patients with RA show an increased sensibility to noxious stimulation (hyperalgesia) at both disease-affected and—non-affected regions (Wendler et al. 2001; Leffler et al. 2002; Edwards et al. 2009). RA is characterized from an altered pain processing in the central nervous system, which is suggested through the typical for this disease autonomic dysfunction and bilateral involvement (Edwards et al. 2009). Additionally, peripheral sensitization of primary afferent nociceptive neurons in inflamed sites plays an important role in the development of pain symptoms. Responsible for this sensitization are various proinflammatory and nociceptive molecules such as cytokines, prostaglandin, neuropeptides and bradykinin. These components are locally produced through inflamed tissue and initiate the process of pain transmission (Sprott 2008).

On the other hand, acute pain has been linked to hyperactivity of the sympathetic nervous system (SNS) (Pickering 2003). Particularly, pain stimuli can lead to a significant increase of both muscle sympathetic nerve activity (MSNA) and blood pressure (Fagius et al. 1989; Nordin and Fagius 1995). However, MSNA has been shown to associate independently of blood pressure with aortic stiffness in healthy individuals (Swierblewska et al. 2010). SNS-activation might promote arterial wall changes (fibrosis, growth of vascular muscles), which lead to an increase of arterial stiffness (Swierblewska et al. 2010). Moreover, SNS can cause an increase of aortic stiffness through its interaction with the renin-angiotensin-aldosterone system (Mancia et al. 2006). Finally, SNS-hyperactivity has been linked to endothelial dysfunction (Sverrisdóttir et al. 2010), which although distinct in terms of pathophysiology, cannot be completely separated from arterial stiffness (Nigam et al. 2003).

Summarizing, there might be a pathophysiological link between pain and the development of aortic stiffness through the effects of increased MSNA on the aortic vasculature.

Until now, the relationship between aortic stiffness and pain has been examined in studies which however included patients without the burden of a systemic inflammatory rheumatic disease (Lee et al. 2011; Kim et al. 2010; Vizzardi et al. 2014; Jin et al. 2015). Interestingly, female patients with fibromyalgia syndrome had statistically higher values of brachial ankle pulse wave velocity (baPWV, a marker of peripheral arterial stiffness) in comparison to their healthy counter partners (Lee et al. 2011). The authors implied a catecholamine associated activation of the autonomic nervous system, which led to an increase of arterial stiffness through endothelial impairment (Wittstein et al. 2005). The same marker was also found to be increased in patients with lumbar disc herniation (Jin et al. 2015). According to the authors, one of the main reasons for the increase of arterial stiffness in those patients was reduced physical activity.

Furthermore, increased augmentation index (indirect marker of arterial stiffness) and baPWV as well as endothelial dysfunction have been reported in patients with migraines (Jiménez Caballero and Muñoz Escudero 2013; Ikeda et al. 2011). Nevertheless, in both studies the increase of arterial stiffness was not linked to pain and its pathophysiological effects. Particularly, in the article of Jiménez Caballero and Muñoz Escudero (2013) the qualitative reduction and loss of function of endothelial progenitor cells, was among others discussed as a cause of impaired endothelial function in patients with migraine (Lee et al. 2008).

### RA-associated parameters and aortic stiffness

Our analysis showed higher mean cfPWV values in the RA group in comparison to the control group. The results however, did not remain statistically significant after controlling for possible confounding factors. Other researchers found cfPWV to be increased in patients with RA in comparison to healthy individuals (Mäki-Petäjä et al. 2006; Kocabay et al. 2012; Turkyilmaz et al. 2013). On the other hand, cfPWV was not found to be statistically different in a population of RA patients who were free of CV risk factors when compared to a group of age and gender matched healthy controls (Arida et al. 2015). Furthermore, in another study cfPWV was found to be only statistically marginally increased in patients with RA in comparison to controls after adjustment for confounding factors (Stamatelopoulos et al. 2009). These conflicting data could be due to heterogeneity of study protocols regarding the amount, age and CV risk factors of examined RA patients and control subjects. A further explanation could be provided through differences in administered immunosuppressant therapies throughout the years, since new RA directed biological drugs lead to a reduction of arterial stiffness (Mäki-Petäjä et al. 2006; Galarraga et al. 2009; Protogerou et al. 2011; Vassilopoulos et al. 2015).

Our exploration did not reveal statistically significant correlations between cfPWV and anti-CCP-antibodies, RF, ANA or disease duration. The presence of anti-CCP-antibodies has been shown to correlate with subclinical vasculopathy (Szekanecz et al. 2007). Anti-CCP-positive-patients show an increase of carotid intima media thickness (cIMT), which is as a marker of subclinical arteriosclerosis (Gerli et al. 2007; Arnab et al. 2013). Furthermore, positivity for anti-CCP is described to be a risk factor for the occurrence of an ischemic cardiac disease (Arnab et al. 2013). Even though anti-CCP-positive patients possess a higher CV risk, there are, to our knowledge, no data to support a direct correlation of this antibody with cfPWV (Pieringer et al. 2010; Provan et al. 2011). The presence of RF has been shown to associate with higher cIMT values and endothelial dysfunction in patients with RA (Rojas-Villarraga et al. 2008; Sahari et al. 2014), but not directly with aortic stiffness (Mäki-Petäjä et al. 2006). We are not aware of studies who examined the relationship between ANA and cfPWV. Finally, our findings regarding the missing association between aortic stiffness and RA disease duration are in agreement with other RA/cfPWV studies (Mäki-Petäjä et al. 2006; Provan et al. 2011; Li et al. 2013).

The present exploration has some limitations: First of all, the low number of patients and control subjects may limit the validity of the results. However, a lot of published cfPWV studies included similar amount of subjects. Secondly, the presence of cardiovascular risk factors in both groups may have had a confounding effect on the results. Despite the fact that adjusted statistical analyses for some of the possible confounders were conducted, the outcomes of the study should be interpreted cautiously.

In conclusion, we report for the first time that aortic stiffness can correlate with the count of tender joints in patients with RA. The association between joint tenderness and aortic stiffness, if proven, could influence future treatment decisions in clinical practice. More studies are needed, in order to verify this finding and to fully understand the mechanisms behind it.
